# Serotonergic Psychedelics Rapidly Modulate Evoked Glutamate Release in Cultured Cortical Neurons

**DOI:** 10.1111/jnc.70020

**Published:** 2025-02-28

**Authors:** Aneta Petrušková, Debarpan Guhathakurta, Enes Yağız Akdaş, Bartomeu Perelló‐Amorós, Renato Frischknecht, Eva‐Maria Weiss, Tomáš Páleníček, Anna Fejtová

**Affiliations:** ^1^ Department of Psychiatry and Psychotherapy Universitätsklinikum Erlangen, Friedrich‐Alexander‐Universität Erlangen‐Nürnberg Erlangen Germany; ^2^ National Institute of Mental Health Klecany Czech Republic; ^3^ Third Faculty of Medicine Charles University Prague Czech Republic; ^4^ Department of Biology, Animal Physiology Friedrich‐Alexander‐Universität Erlangen‐Nürnberg Erlangen Germany

**Keywords:** 5‐HT2A, fluorescent sensors, neurotransmitter release, presynaptic, short‐term plasticity, synaptic vesicles

## Abstract

The serotonergic psychedelics psilocybin, LSD and DMT hold great promise for the development of new treatments for psychiatric conditions such as major depressive disorder, addiction and end‐of‐life anxiety. Previous studies in both animals and humans have confirmed the effects of these drugs on neuronal activity and plasticity. However, the understanding of the mechanisms of action of these substances is limited. Here we show rapid effects of psychedelics on presynaptic properties, using live cell imaging at the level of single synapses in primary rat cortical neurons. Using the genetically encoded reporter of synaptic vesicle fusion synaptopHluorin, we detected a reduced fraction of synaptic vesicles that fused in response to mild or strong electrical stimulation 3–30 min after application of serotonergic psychedelics. These effects were transient and no longer present 24 h after treatment. While DMT only reduced the total recycling pool, LSD and psilocin also reduced the size of the readily releasable vesicle pool. Imaging with the sensors for glutamate, iGluSnFR, and presynaptic calcium, synGCaMP6, showed that while psilocin and DMT increased evoked glutamate release, LSD and psilocin reduced evoked presynaptic calcium levels. Interestingly, psilocin also affected short‐term plasticity leading to a depression of responses to paired stimuli. The rapid and drug‐specific modulation of glutamatergic neurotransmission described in this study may contribute to distinct anxiolytic and antidepressant properties of serotonergic psychedelics.

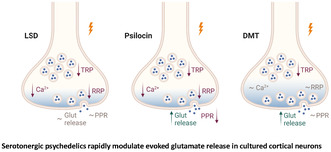

## Graphical abstract

The present study demonstrates that the serotonergic psychedelics LSD, psilocin, and DMT acutely modulate presynaptic neurotransmitter release properties in primary neuronal cultures. All psychedelics reduced the total recycling pool (TRP) of synaptic vesicles, while LSD and psilocin also reduced the readily releasable pool (RRP). LSD and psilocin reduced presynaptic evoked calcium transients. DMT and psilocin increased evoked glutamate release. Interestingly, psilocin also decreased the paired‐pulse ratio (PPR), indicating a short‐term depression of glutamate release evoked by repeated stimulations. These findings reveal substance‐specific regulation of the presynaptic properties and advance the understanding of mechanisms underlying psychedelics' effects.

**Abbreviations**
5‐HT: serotonin
AAV: adeno‐associated virus
AP: action potential
AP5: D‐(‐)‐2‐amino‐5‐phosphonopentanoic acid
BDNF: brain‐derived neurotrophic factor
cAMP: cyclic adenosine monophosphate
CNQX: 6‐cyano‐7‐nitroquinoxaline‐2,3‐dione disodium
CTRL: control (vehicle)
DAPI: 4′,6‐diamidino‐2‐phenylindole
DMSO: dimethyl sulfoxide
DMT: *N,N*‐dimethyltryptamine
GFP: green fluorescent protein
ICC: immunocytochemistry
IF: immunofluorescence iGluSnFR
KTSR: glutamate‐sensitive fluorescent reporter Ketanserin
LIS: lisuride
LSD: lysergic acid diethylamide
NMDAR: *N*‐methyl‐D‐aspartate receptor
pCREB: cAMP response element‐binding protein
PKA: protein kinase A
PSI: psilocin
qRT‐PCR: quantitative real‐time polymerase chain reaction ROI
RRID: Region of interest Research Resource Identifier
RRP: readily releasable pool
SEM: standard error of mean
SER: serotonin
SERT: serotonin transporter
SV: synaptic vesicle
sypHy: synaptopHluorin
TRP: total recycling pool

## 1 Introduction

Serotonergic psychedelics are drugs that induce altered states of consciousness. Over the past decade, there has been a considerable increase in interest in psychedelic drugs due to their potential therapeutic efficacy for psychiatric conditions, including major depressive disorder, addiction, post‐traumatic stress disorder, and end‐of‐life anxiety. Among the potential benefits of psychedelics are their rapid onset of action, which occurs within hours after treatment, and their long‐lasting effects, which can last for months after one or two sessions (Goodwin et al. 2023, 2022; Kopra et al. 2023; Galvão‐Coelho et al. 2021; Andersen et al. 2021; Gregorio et al. 2021; Carhart‐Harris et al. 2016, 2018). The mechanisms of action of these drugs are not well understood, and further research is needed, particularly given the potential for their use in psychiatric disorders, where current first‐line treatments have a long lag time and high rates of treatment resistance.

This study focuses on the serotonergic psychedelics (further mentioned as psychedelics) lysergic acid diethylamide (LSD), *N,N*‐dimethyltryptamine (DMT), an active compound of the Amazonian indigenous brew ayahuasca (Franzen and Gross 1965); and psilocin, the active metabolite of psilocybin found in the *Psilocybe* mushrooms. These drugs have a similar structure to serotonin (5‐hydroxytryptamine, 5‐HT) and act predominantly as 5‐HT receptor (5‐HTR) agonists. Psilocin is a partial agonist of the 5‐HT2AR, but also binds with a similar Ki (in the order of tens of nM) to other serotonergic receptors including the 5‐HT1A/B/D/E, 5‐HT2C, 5‐HT5A, 5‐HT6 and 5‐HT7, while having lower affinity to 5‐HT2B (Ki in the order of hundreds of nM) (Besnard et al. 2012; PDSP 2023; Rickli et al. 2016). DMT also acts as a partial 5‐HT2A agonist (Ki in the order of hundreds nM) and has comparable affinities for other 5‐HT (5‐HT1A, 5‐HT1D, 5‐HT1E, 5‐HT2B, 5‐HT2C, 5‐HT5, 5‐HT6 and 5‐HT7) and dopaminergic D1 receptors. Additionally, it displays lower affinities for 5‐HT1B, sigma‐1 receptors and SERT (Fontanilla et al. 2009; PDSP 2023). LSD exhibits the most complex binding profile, with the highest affinity for the 5‐HT2A/C, 5‐HT1A, 5‐HT5A/B, 5‐HT6 and 5‐HT7 receptors (Ki in the order of units of nM), followed by the 5‐HT2B, 5‐HT1D/E and 5‐HT1B receptors. LSD also binds dopaminergic (particularly D2 and D3, with binding affinities in the order of tens of nM), adrenergic (hundreds of nM), and with even lower affinity also to TAAR and histamine 1 receptor (PDSP 2023; Ray 2010). For all serotonergic psychedelics, 5‐HT2AR agonism has been proposed to play a pivotal role in the acute perceptual, cognitive and behavioral effects (Holze, Vizeli, et al. 2021; Jaster et al. 2022; Preller et al. 2017; Stenbæk et al. 2021; Vollenweider et al. 1998). In addition, the 5‐HT1A receptors have been linked to some of the behavioral effects of LSD and psilocin (Halberstadt et al. 2011; Krebs‐Thomson and Geyer 1996). Experiments with cultured rodent neurons showed psychedelics‐induced alterations in gene expression, neuronal outgrowth, synapse formation, and synaptic plasticity, which were dependent on the activation of the 5‐HT2AR (Ly et al. 2018). However, other studies reported antidepressant and anxiolytic behavioral effects after psychedelics, which were independent of 5‐HT2 receptors (Hesselgrave et al. 2021; Moliner et al. 2023). Thus, the debate on the role of the 5‐HT2AR in mediating the beneficial neuroplasticity induced by psychedelics is still ongoing.

It is anticipated that psychedelics will modulate synaptic transmission by acting on synaptic neurotransmitter receptors. The activation of 5‐HT receptors by their endogenous ligand serotonin has been demonstrated to alter the phosphorylation status and abundance of synaptic vesicle‐associated proteins, the dynamics of synaptic vesicles (SVs), and the release of neurotransmitters (Patzke et al. 2019). It has been previously shown that serotonin is capable of both increasing and decreasing neuronal activity through the action of 5‐HT2A and 5‐HT1A/B receptors, respectively (Aghajanian and Marek 1999; Singer et al. 1996; Tanaka and North 1993). This is due to the differential coupling of individual classes of 5‐HT receptors to specific downstream signalling cascades, resulting in a broad spectra of 5‐HT‐induced cellular responses.

The potential effect of psychedelics on neurotransmitter release from presynaptic terminals is a topic of considerable interest, particularly in light of recent findings suggesting a comparable effect for the novel, rapid‐acting antidepressant ketamine and its metabolite hydroxynorketamine (Guhathakurta et al. 2024; Lazarevic et al. 2021). Ketamine is a non‐competitive NMDAR antagonist that is rapidly metabolised in the body to hydroxynorketamine, which has also been demonstrated to elicit antidepressant‐like effects in animal models (Li et al. 2010; Zanos et al. 2016). However, the precise molecular targets remain unclear. A recent study showed that although each drug binds to distinct receptors, the downstream molecular cascades converge to reduce the fusion competence of synaptic vesicles and glutamate release and activate the nuclear transcription factor CREB, which is crucial for antidepressant‐related changes in gene expression (Guhathakurta et al. 2024). Therefore, drug‐induced regulation of the presynaptic function presents a plausible mechanism of action for rapid‐acting antidepressants.

In order to address the question of presynaptic regulation by serotonergic psychedelics, we investigated the effects of LSD, DMT, and psilocin on evoked glutamate release. Specifically, we performed live‐cell imaging with genetically encoded sensors to directly observe synaptic vesicle fusion, evoked glutamate release and presynaptic calcium transients at the level of individual synapses in cultured rat cortical neurons. Furthermore, we compared the effects of psychedelics on SV fusion with serotonin receptor agonists and antagonists, with the aim of identifying synaptic receptors that may mediate the presynaptic effects of psychedelics.

## 2 Materials and Methods

### 2.1 Animals

For preparation of primary cultures, E18 Sprague Dawley rats (RjHan: SD) obtained from Janvier Labs were used. Experiments were carried following the European Council Directive (2010/63/EU, amendment 2019), fully in accordance with local regulations, registered under local TS13/2016 and approved by the authorities Regierung Unterfranken (55.2.2‐2532‐2‐1802).

### 2.2 Antibodies

Antibodies used for immunocytochemistry experiments: phospho‐CREB (Ser133; 1:700, Cell Signaling Technology, RRID:AB_2561044) and MAP2 (1:500, Sigma‐Aldrich, RRID:AB_477193). Secondary antibodies: Cy3 AffiniPure Donkey Anti‐Rabbit (RRID:AB_2307443), Cy5 AffiniPure Donkey Anti‐Mouse (RRID:AB_2340819) (both 1:1000, Jackson ImmunoResearch).

### 2.3 Drugs and Chemicals

For treatment of cells, the following drugs were used: lysergic acid diethylamide (LSD) fumarate (custom synthesis, Alfarma), *N,N*‐dimethyltryptamine (DMT) fumarate (cat. no. 9003568, Cayman Chemical), 4‐hydroxydimethyltryptamine (psilocin, cat. no. 11864, Cayman Chemical), all diluted in 5% DMSO; 5‐HT2AR antagonists M100907 (volinanserin; cat. no. T5389, TargetMol) diluted in DMSO and ketanserin tartrate (cat. no. AG‐CR1‐0014‐M010, AdipoGen Life Sciences) in 2.5% DMSO, serotonin hydrochloride (cat. no. H9523, Sigma‐Aldrich) in H_2_O, lisuride maleate (cat. no. 4052, Tocris), 5‐HT7 rec. antagonist DR4485 hydrochloride (cat. no. 5005, Tocris) in 2% DMSO and 5‐HT1A rec. antagonist NAD299 hydrochloride (cat. no. 3282, Tocris) in H_2_O. All drugs were diluted in DMSO and/or water and kept in aliquots at −80°C for storage or −20°C for short‐term use. Maximal final concentration of DMSO in cell media after treatment or solution used for imaging was 0.05%. For sypHy live cell imaging, D‐(‐)‐2‐Amino‐5‐phosphonopentanoic acid (AP5, cat. no. 0106, Tocris), 6‐Cyano‐7‐nitroquinoxaline‐2,3‐dione disodium (CNQX, cat. no. 0190, Tocris) and bafilomycin A1 (cat. no. S1413, Selleck) were used.

### 2.4 Primary Rat Cortical Cultures

Primary cortical cultures from rat E18 embryos were prepared as previously described (Anni et al. 2021). Briefly, the pregnant rats were subjected to inhalation anesthesia with 4% isoflurane with oxygen as the carrier gas and decapitated using guillotine. The embryos were rapidly isolated and precisely decapitated, and brains were freed from skull and meninges in ice‐cold HBSS‐/‐ (cat. no. 14175053), cortices isolated and pooled, followed by incubation with 0.25% trypsin (cat. no. 15400054, both Thermo Fisher Scientific) at 37°C for 20 min. Tissues were then triturated using syringes with injection needles 0.90 and 0.45 mm in diameter in a solution of 0.1 mg/mL DNase I (cat. no. 11284932001, Roche), and the resulting cell suspension was filtered through a 100 μm nylon cell strainer. Cells were seeded in DMEM (cat. no. 41966029, Thermo Fisher Scientific) containing 10% fetal calf serum (FCS, cat. no. S0015, Biochrom), 2 mM L‐Glutamine (cat. no. 25030024) and 1% Antibiotic/antimycotic (cat. no. 15240062, both Thermo Fisher Scientific) in a density of 200,000 cells in 1 mL per coverslip (18 mm Menzel glass in 12‐well plates coated with 0.5 mg/mL poly‐L‐lysine [cat. no. P1524, Sigma‐Aldrich]). After 1 h allowing cells to attach, the medium was replaced with Neurobasal (cat. no. 12348017) supplemented with 2% B27 (cat. no. 17504044), 0.8 mM GlutaMAX (cat. no. 35050‐038) and 1% Antibiotic/Antimycotic (all from Thermo Fisher Scientific). Cultures were maintained in a 5% CO_2_ containing humidified incubator at 37°C until the experimental date and fed with fresh media once a week.

### 2.5 Quantitative Immunocytochemistry

Mature neuronal cultures at DIV20‐23 were fixed after treatment with drugs in 4% paraformaldehyde for 4 min at room temperature (RT). After washing with PBS, cells were blocked and permeabilized with 10% FCS, 0.3% TritonX‐100 and 0.1% glycine in PBS for 45 min and incubated with primary antibody solution at 4°C overnight. The next day, cells were washed with PBS and incubated for 1 h with corresponding secondary antibodies at RT, washed with PBS and water and mounted on slides using Fluoroshield with (cat. no. F6057) or without DAPI (cat. no. F6182, Sigma‐Aldrich). Antibodies were diluted in PBS solution containing 3% FCS. All experimental conditions compared in one experiment were processed in parallel using identical solutions.

### 2.6 Image Acquisition and Analysis

Images or videos of each individual experiment were acquired within 1 day with identical settings of illumination and camera for all samples of that experiment. For all image acquisition, we used a Nikon Eclipse Ti epi‐fluorescence microscope equipped with an iXon EM+ 885 EMCCD Andor camera, 60X/NA1.2 (for live‐cell imaging; CFI Plan Apo VC WI ∞/0.15–0.18 DIC N2 WD 1.0 objective, Nikon) and 20X/0.75NA (for pCREB staining; CFI Plan Apo VC AI ∞/0.17 WD 1.0 objective, Nikon) controlled by VisiView software (Visitron Systems, RRID:SCR_022546) and illuminated by Omicron LedHUB (Omicron‐Laserlage Laserprodukte). For ICC, 6–13 visual fields from each of 2 coverslips were acquired for every condition within one experiment. ImageJ was used for processing and analyzing images and videos. For pCREB ICC image analysis, a spherical ROI was placed over DAPI‐stained nuclei of MAP2 positive cells (10–15 nuclei per image), transposed to the pCREB staining channel, where the average immunofluorescence (IF) intensity was measured for each nucleus. Average background intensities of respective pictures were subtracted from nuclear intensity. Mean IF intensities were then normalized to the mean IF intensities of the control group of the same experiment.

### 2.7 Production of Viral Particles and Transduction

SypHy sensor originally described by (Rose et al. 2013) was expressed from a lentiviral vector as previously described in (Lazarevic et al. 2017). Lentiviral particles were produced in HEK293T cells (RRID: CVCL_0063) using FuGENE HD Transfection Reagent (cat. no. E2311, Promega) according to the manufacturer's instructions. Briefly, FUGW‐based sypHy vector, psPAX2 (RRID:Addgene_12260) and pVSVG (RRID:Addgene_8454) packaging and pseudotyping vectors were used for transfection of 90% confluent HEK293T cells at a concentration of 1.64: 0.72: 1.3 (in pmol), respectively, in DMEM media supplemented with 10% FCS, 2 mM L‐glutamine, and 1% Antibiotic/Antimycotic. This media was replaced 16 h after transfection by Neurobasal (supplemented as mentioned above). 24 and 48 h later, cell media was collected, centrifuged at 500 g for 5 min, and the supernatant containing lentiviral particles was stored in single‐use aliquots at −80°C.

Adeno‐associated viruses (AAVs) were used to express the genetically encoded glutamate sensor SF.iGluSnFR.S72A (Marvin et al. 2019) and the presynaptic calcium sensor synGCaMP6 (Müller et al. 2022). To produce AAVs, HEK293T cells were transfected with the plasmids of pAAV‐DJ, pHelper and pAAV.hSynapsin.SF‐iGluSnFR.S72A or rAAV‐Syn‐Synaptophysin‐GCamp6 in a 1:1:1 ratio (30 μg for T‐75) using Fugene HD transfection reagent. Following 3 days in culture, cells were centrifuged and the supernatant collected. The cell pellet was resuspended in 20 mM Tris and 150 mM NaCl buffer, and freeze‐thawing by liquid nitrogen was used to lyse the cells. After another centrifugation for removing cell debris, the supernatant was collected and mixed with the previous stock of supernatant. The solution was incubated overnight at 4°C with PEG‐8000/0.5 M NaCl in order to precipitate the viral particles. Finally, the solution was centrifuged at 2500*g* for 1 h and the pellet of viral particles was resuspended in TNE buffer (100 mM Tris, pH = 8.0, 150 mM NaCl, 20 mM EDTA), aliquoted and stored at −80°C. pAAV.hSynapsin.SF‐iGluSnFR.S72A was a gift from Loren Looger (RRID:Addgene_106176) and rAAV‐Syn‐Synaptophysin‐GCamp6 derived from GCaMP6f was a kind gift of Susanne Schoch (Müller et al. 2022).

### 2.8 SynaptopHluorin (sypHy) Imaging and Analysis

Neuronal cultures were transduced with lentiviral particles for the expression of sypHy at DIV2‐3 and imaged at DIV20‐27. Coverslips were transferred to the imaging chamber with field stimulation electrodes (cat. no. RC‐49MFSH, Warner Instruments). Stimulus isolator A 385 (World Precision Instruments) connected to a stimulus generator (cat. no. STG‐4008, Multi‐Channel Systems) was used to induce electric stimulation. Cells were imaged in the presence of Tyrode's buffer (TB; 2.5 mM KCl, 25 mM HEPES, 30 mM glucose, 2 mM CaCl_2_, 2 mM MgCl_2_, 119 mM NaCl, pH = 7.4) containing 10 μM CNQX and 50 μM AP5 to prevent recurrent network activity, and 1 μM Bafilomycin A1 to avoid vesicle re‐acidification upon endocytosis of released vesicles. Imaging was carried out using the microscope setup described above at RT with the Cy3 filter set (in nm: excitor 543/22 and emitter 593/40) for the visualization of transduced neurons, and the GFP filter set (in nm: excitor 472/30 and emitter 520/35) for imaging of SV fusion at the rate of 1 frame/s. The imaging protocol consisted of a baseline recording of 15 s followed by stimulation of 40 action potentials (APs) at 20 Hz to release the readily releasable pool (RRP), and 900 APs at 20 Hz to release the total recycling pool (TRP). Finally, 60 mM NH_4_Cl was applied to alkalize and therefore unquench all sypHy‐expressing vesicles (Burrone et al. 2006). A single recording was acquired from each coverslip.

Analysis of imaging data was carried out in ImageJ (National Institutes of Health, RRID:SCR_003070) using the SynQuant plugin (Wang et al. 2020). Time lapse stack series were first cropped, and responding synaptic puncta were visualised by maximal intensity z‐projection of 70 frames following 900 AP stimulation, from which maximal intensity z‐projection of baseline (frames 3–13) was subtracted, so that only active synapses were considered for analysis. The SynQuant plugin was then used for automated detection of synaptic puncta (approximately 100–200 in each recording), and its intensity was quantified in all images of the time lapse, which was then averaged to get a single trace of fluorescence intensity for each recording. Bleaching correction was applied by normalising the data by bleaching factor calculated from recording without stimulation. Average background intensity was subtracted for each recording. Finally, fluorescence traces were normalised to minimum (baseline; *F*_min_) – maximum (NH_4_Cl; *F*_max_) signal using formula $\left(F-F_{\min}\right)/\left(F_{\max}-F_{\min}\right)$. The sizes of RRP (measured from frames 23–37) and TRP (frames 130–159) were quantified as relative fractions of the total sypHy‐expressing pool (NH_4_Cl pulse; *F*_max_).

### 2.9 iGluSnFR And synGCaMP6 Imaging and Analysis

Cortical cultures were transduced with AAVs for the expression of iGluSnFR or synGCaMP6 on DIV10‐14 and imaged after maturation on DIV24‐26 following a 30 min treatment with drugs. The same microscope and stimulation setup was used as mentioned above. Cells were imaged in the presence of Tyrode's buffer (TB) containing 10 μM CNQX and 50 μM AP5 at RT (TB solution as above or in case of 1 mM Ca^2+^ experiments modified as follows: 2.5 mM KCl, 25 mM HEPES, 30 mM glucose, 1 mM CaCl_2_, 2 mM MgCl_2_, 125 mM NaCl, pH = 7.4). To analyze glutamate release, iGluSnFR responses were recorded elicited by paired pulses (PP) spaced by 100 ms delivered after a 6 s long baseline recording and repeated three times in a given visual field with a 10 s recovery phase between stimulations. Not more than 6 recordings were acquired from different regions of one coverslip with an acquiring frequency of 60 frames/s. For imaging of synGCaMP6, baseline (15 s) and responses to 5 single stimuli (at 0.1 Hz) and a final burst stimulation of 30 APs at 40 Hz were acquired at 15 frames/s. For the paired‐pulse, baseline (15 s) and responses to paired stimuli (with 100 ms interval) delivered five times at 0.1 Hz were at 83 frames/s.

Time‐lapse series were processed in ImageJ using the SynQuant plugin for synapse detection, similarly to sypHy imaging. For iGluSnFR: active glutamate release sites were detected from the maximum intensity z‐projection of the first PP stimulation with SynQuant. Intensity of detected puncta (30–80 per visual field) was measured throughout each time‐lapse series and averaged to get a single trace. Average baseline intensity (from 50 frames preceding each PP) was subtracted from the amplitude of the first peak, and the lowest value between the two peaks was subtracted from the second peak in order to account for the signal accumulation. The ratio of the second peak (P2) to the first peak (P1) was then calculated. Ratios from the stimulations in one recording were averaged to obtain a single value per recording. For synGCaMP6: same procedure was used for analysis as for iGluSnFR, with the exception that the z‐projection was created from all frames between the first and last stimulation. There were between 150 and 250 detected puncta per visual field. Only amplitudes of fluorescence intensity after all stimulation types were measured (including paired‐pulse stimulation) and were averaged to produce a single value per stimulation type per recording.

### 2.10 Statistical Analysis

Statistical analysis of all data was performed in Prism 9 (GraphPad Software, RRID:SCR_002798). Utilised statistical tests include Student's *t*‐test, one‐way ANOVA followed by Dunnett's multiple comparisons test, and Kruskal–Wallis test with Dunn's multiple comparisons test (for data sets which did not follow normal distribution). Two‐way ANOVA with Šídák's multiple comparisons test was used for the analysis of paired‐pulse ratio combining 1 mM and 2 mM Ca^2+^ data sets. The choice of specific test is indicated in the figure legends for each experiment, together with group size and statistical significance, and is marked as * for *p* < 0.05, ** for *p* < 0.01 and *** for *p* < 0.001 in all plots. The grouping of treatments for statistical testing was dependent on the experimental setup, that is, treatments that were processed during an experiment together were analyzed together. Assumptions of statistical tests (i. e. normal distribution and distribution of variance) were tested for each data set, and appropriate tests were then applied accordingly, as described in the figure legends. The alpha level used for analyzing significance was 0.05. No tests for outliers were performed in order to exclude data points.

## 3 Results

### 3.1 Serotonergic Psychedelics Increase Nuclear Localization of pCREB After 24 h Treatment, but Not at Earlier Time Points

**FIGURE 1:**
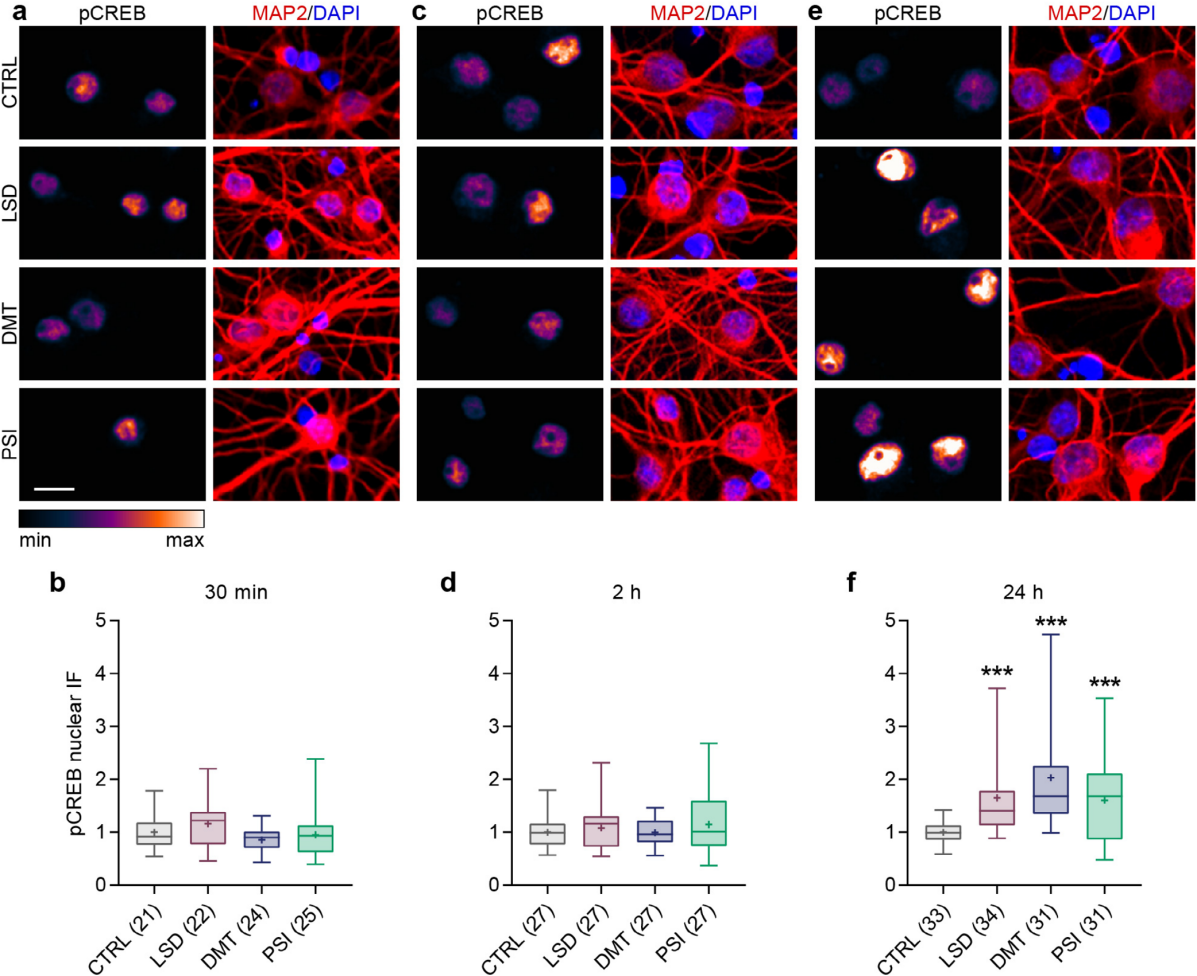
Serotonergic psychedelics increase nuclear localization of phosphorylated CREB after 24 h treatment. Representative images of immunofluorescent detection of pCREB Ser133 (pseudocoloured, range shown in the rectangle below) and dendritic marker MAP2 (red) overlaid with DAPI nuclear staining (blue) in mature cortical cultures treated with CTRL or drugs for 30 min (a), 2 h (c) or 24 h (e). Scale bar is 15 μm. Quantification of nuclear immunofluorescence (IF) of pCREB after treatment with CTRL or drugs for 30 min (b), 2 h (d) or 24 h (f), number of visual fields (each consisting of 10–15 analysed nuclei) from 2 to 3 independent experiments in brackets. Data did not pass Shapiro–Wilk test for normality, and were assessed by Kruskal–Wallis test (30 min: *H*_(3,92)_ = 7.020, *p* = 0.071; 2 h: *H*_(3,108)_ = 1.003, *p* = 0.801; 24 h: *H*_(3,128)_ = 40.26, *p* < 0.001) and Dunn's multiple comparisons test of CTRL versus each drug (24 h: *p* < 0.001 for all comparisons).

Psychedelics have been shown to induce long‐lasting alterations in neuronal structure, gene expression patterns and animal and human behaviour. Our initial objective was to ascertain whether psychedelics, when administered at concentrations previously employed, could elicit neuroplasticity‐like alterations in our experimental setting. Therefore, we tested treatment‐induced changes in the nuclear abundance of phosphorylated CREB (pCREB). pCREB is the major regulator of activity‐dependent transcription, controlling the expression of numerous genes, including brain‐derived neurotrophic factor (BDNF), which is essential for psychedelic‐induced neuroplasticity (Lonze and Ginty 2002; West et al. 2002). Therefore, mature cultured cortical neurons (DIV 20–23) were treated with LSD (10 μM), DMT (90 μM) and psilocin (PSI; 10 μM) or vehicle for 30 min, 2 h or 24 h. The effect of treatment on the activation of transcription factor CREB was assessed by immunostaining with a phospho‐specific antibody against the active CREB, phosphorylated at its Ser133. MAP2 and DAPI staining was done to identify neuronal somata and nuclei, respectively. The activation of CREB was assessed by quantification of the pCREB immunofluorescence (IF) within neuronal nuclei (Figure 1a,c,e). We detected no changes in pCREB IF 30 min or 2 h after the application of drugs (Figure 1a–d). However, an increase in nuclear pCREB IF was evident 24 h after the treatment, as compared to CTRL (Figure 1e,f; mean ± SD: CTRL: 100% ± 21%, LSD 165% ± 72%, DMT 213% ± 109%; PSI 160% ± 80%). This confirms that the drugs at the used concentrations induce nuclear CREB activation in our experimental system.

### 3.2 Serotonergic Psychedelics Decrease Synaptic Vesicle Fusion Competence Following Short‐Term Treatment, but Not After 24 h

To examine the effect of psychedelics on the evoked neurotransmitter release, we monitored the evoked fusion of SV using a pH‐sensitive fluorescent probe synaptopHluorin‐tdimer2 (sypHy) expressed by rat cortical neurons. SypHy is derived from the integral SV protein synaptophysin and contains the pH‐sensitive GFP inserted into its luminal domain. In the acidic milieu of the SV, the fluorescence of sypHy is quenched, but rapidly increases when the SV fuses with the neuronal membrane exposing sypHy to the neutral pH of the extracellular solution. The cells were stimulated electrically using field electrodes. First, 40 action potentials (APs) at 20 Hz were applied to induce fusion of SVs docked at the presynaptic membrane (the readily releasable pool, RRP), followed by 900 APs at 20 Hz to induce fusion of all fusion‐competent SVs (the total recycling pool, TRP) (Burrone et al. 2006). At the end of the recording, NH_4_Cl was applied to unquench all sypHy within SVs, allowing calculation of the size of the RRP and the TRP relative to all vesicles present at each synapse.

**FIGURE 2:**
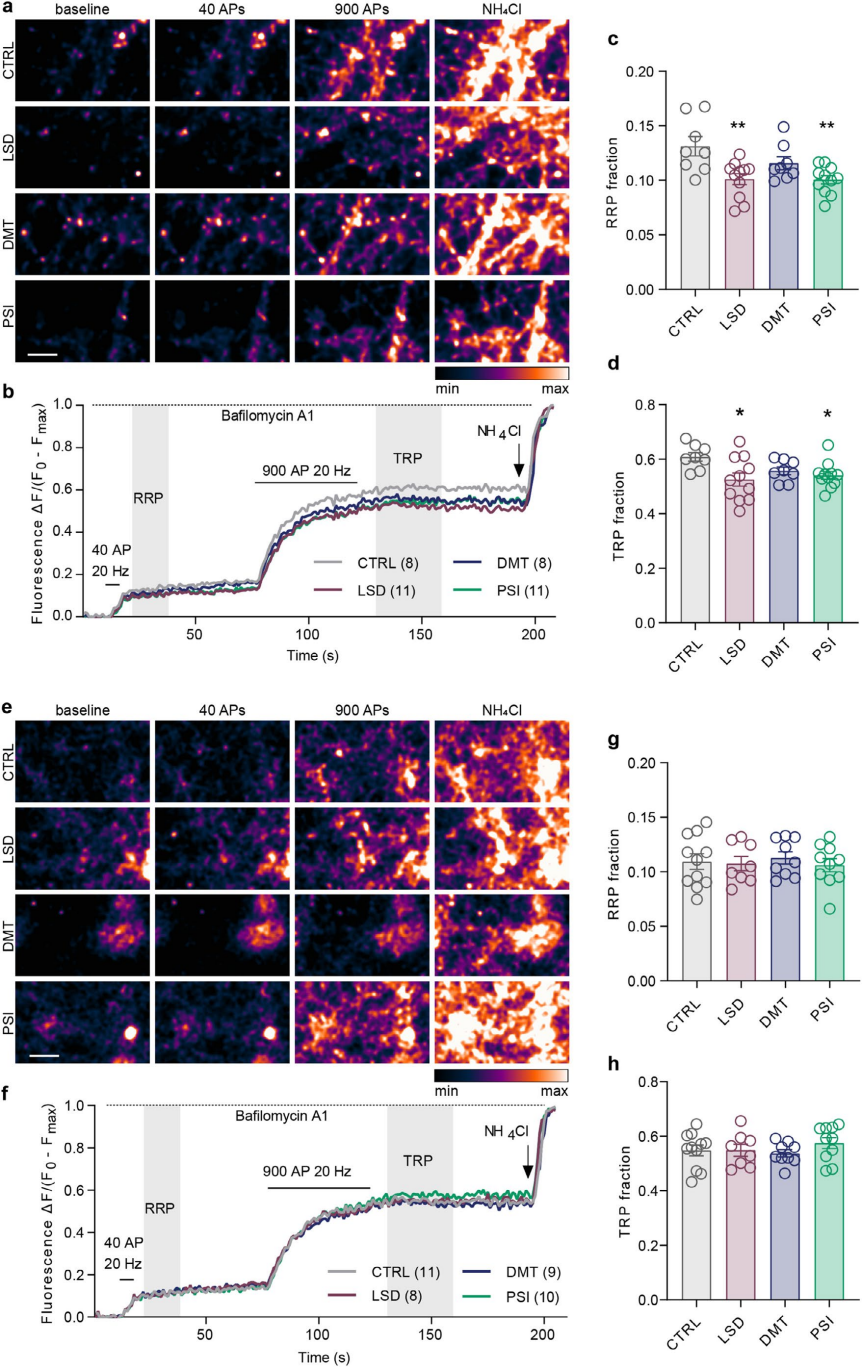
LSD and psilocin reduce the fusion competence of SVs after 30 min but not after 24 h treatment. Representative colour gradient (min to max range shown in the rectangle below) images from live‐cell imaging of sypHy‐expressing primary cortical cultures treated with vehicle (CTRL), LSD, DMT and PSI for 30 min (a) or 24 h (e) during baseline recording, stimulation with 40 and 900 APs at 20 Hz, and upon NH_4_Cl pulse, in the presence of bafilomycin A1. Scale bars are 10 μm. (b, f) Average traces of sypHy fluorescence intensity from (a, e), respectively, normalised to minimum (baseline, *F*_0_) and maximum (NH_4_Cl pulse, *F*_max_). Total number of recordings (independent coverslips) per treatment from 3 to 4 independent culture preparations indicated in brackets. Quantification of the RRP (c, g) and TRP (d, h) fractions of SVs from the respective experiments. In graphs, bars show the mean, whiskers SEM, and circles all data points. Statistical significance assessed by one‐way ANOVA (30 min: RRP: *F*_(3,34)_ = 6.071, *p* = 0.002; TRP: *F*_(3,34)_ = 3.512, *p* = 0.026; 24 h: RRP: *F*_(3,34)_ = 0.189, *p* = 0.905; TRP: *F*_(3,34)_ = 0.694, *p* = 0.562) and Dunnett's multiple comparisons test CTRL versus each drug (30 min: RRP: LSD *p* = 0.002, DMT *p* = 0.205, PSI *p* = 0.002; TRP: LSD *p* = 0.010, DMT *p* = 0.192, PSI *p* = 0.040).

LSD (10 μM), DMT (90 μM), psilocin (PSI; 10 μM) or vehicle was applied to the media of mature cortical neurons 30 min prior to imaging. While DMT had no effect, we observed a significant reduction in the RRP as well as in the TRP in cells treated with LSD and PSI (Figure 2a–d; RRP: CTRL 0.13 ± 0.02, LSD 0.10 ± 0.02, DMT 0.12 ± 0.02, PSI 0.10 ± 0.01; TRP: CTRL 0.61 ± 0.05, LSD 0.53 ± 0.08, DMT 0.56 ± 0.04, PSI 0.54 ± 0.05). The observed effect was transient for all drugs. No effects were detected when synaptic vesicle pools were analyzed 24 h after treatment (Figure 2e–h; RRP: CTRL 0.11 ± 0.02, LSD 0.11 ± 0.02, DMT 0.11 ± 0.02, PSI 0.11 ± 0.02; TRP: CTRL 0.55 ± 0.07, LSD 0.55 ± 0.06, DMT 0.54 ± 0.04, PSI 0.58 ± 0.06).

**FIGURE 3:**
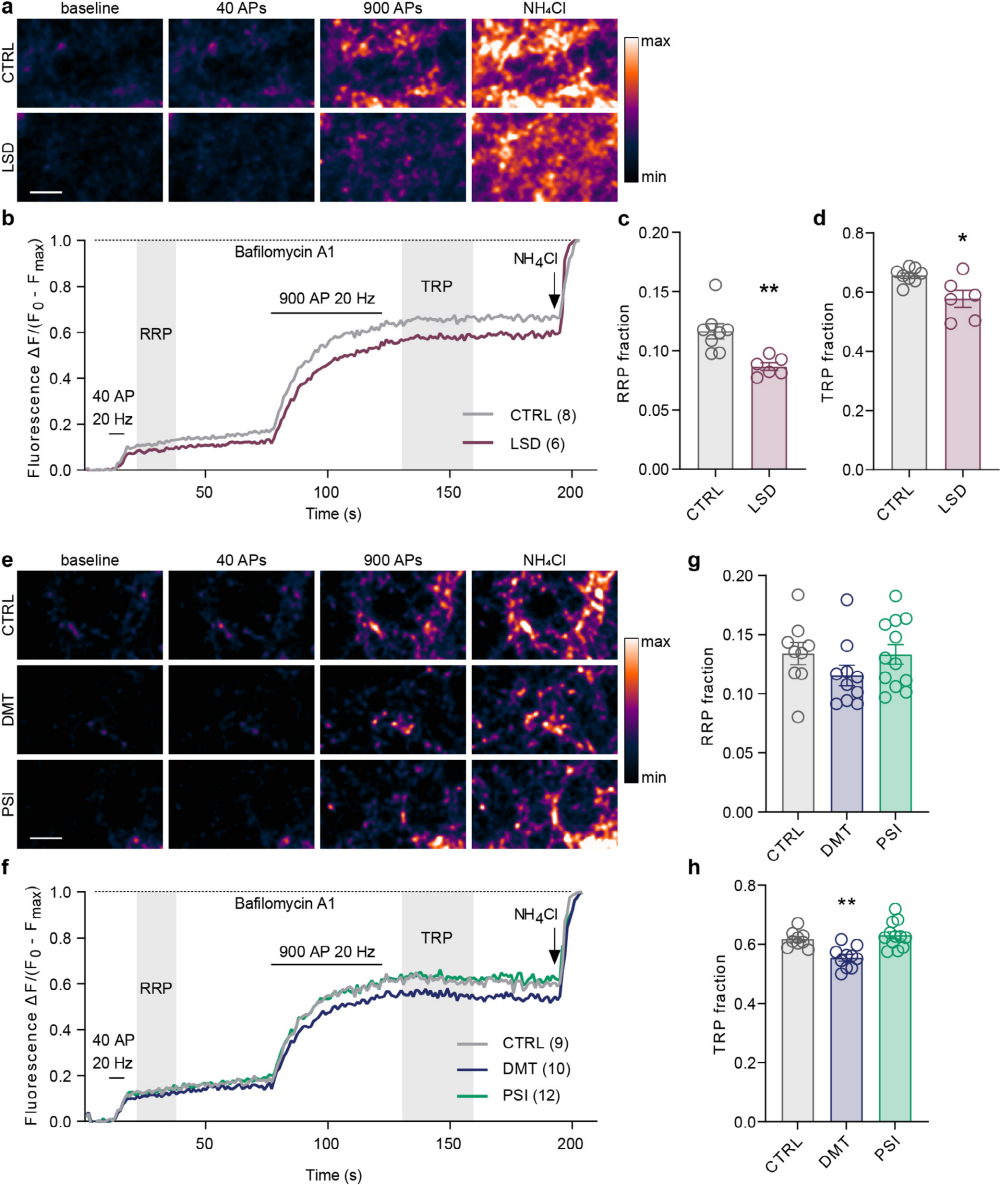
LSD and DMT, but not psilocin, reduce fusion competence of SVs upon acute treatment. (a, e) Representative colour gradient images (min to max range is shown in the rectangle below) from live‐cell imaging of sypHy‐expressing primary cortical cultures treated with vehicle (CTRL) and LSD (a), or CTRL, DMT and PSI (e). Drugs were added to the imaging solution immediately before (2–3 min) recording. SV fusion was evoked by 40 and 900 APs at 20 Hz and upon NH_4_Cl pulse, in the presence of bafilomycin A1. Scale bars 10 μm. (b, f) Average traces of sypHy fluorescence intensity from (a, e) respectively, normalised to baseline (*F*_0_) and NH_4_Cl response (*F*_max_). Total number of recordings (independent coverslips) per treatment from 2 to 3 independent culture preparations indicated in brackets. Quantification of the RRP (c, g) and TRP (d, h). In graphs, bars show the mean, whiskers SEM, and circles all data points. Statistical significance assessed by unpaired *t*‐test for LSD (RRP: *t*_(12)_ = 3.757, *p* = 0.003; TRP: *t*_(12)_ = 2.956, *p* = 0.012) and by one‐way ANOVA (RRP: *F*_(2,28)_ = 1.397, *p* = 0.264; TRP: *F*_(2,28)_ = 12.35, *p* < 0.001) with Dunnett's multiple comparisons test for CTRL versus PSI or DMT (TRP: PSI *p* = 0.612, DMT *p* = 0.002).

Subsequently, the same experiment was conducted, with the addition of the compounds to the imaging solution immediately prior to the start of the recording. This allowed for the examination of the acute effects of the drugs. Acute treatment with LSD significantly decreased both the RRP and TRP (Figure 3a–d; RRP: CTRL 0.12 ± 0.02, LSD 0.09 ± 0.01; TRP: CTRL 0.66 ± 0.03, LSD 0.58 ± 0.07). DMT treatment reduced the TRP, but not RRP, and PSI had no effect on synaptic vesicle pools under these conditions (Figure 3e–h; RRP: CTRL 0.13 ± 0.03, DMT 0.12 ± 0.03, PSI 0.13 ± 0.03; TRP: CTRL 0.62 ± 0.03, DMT 0.56 ± 0.04, PSI 0.63 ± 0.04).

In conclusion, we observed a robust and rapid reduction of the RRP and TRP upon treatment with LSD. Psilocin and DMT exerted specific time‐dependent effects on the RRP and TRP. It is noteworthy that all these effects were transient, disappearing 24 h after treatment.

### 3.3 5‐HT2A Receptor Antagonist Affects RRP and TRP, Whereas 5‐HT7 and 5‐HT4 Receptor Antagonists Only Act on TRP

**FIGURE 4:**
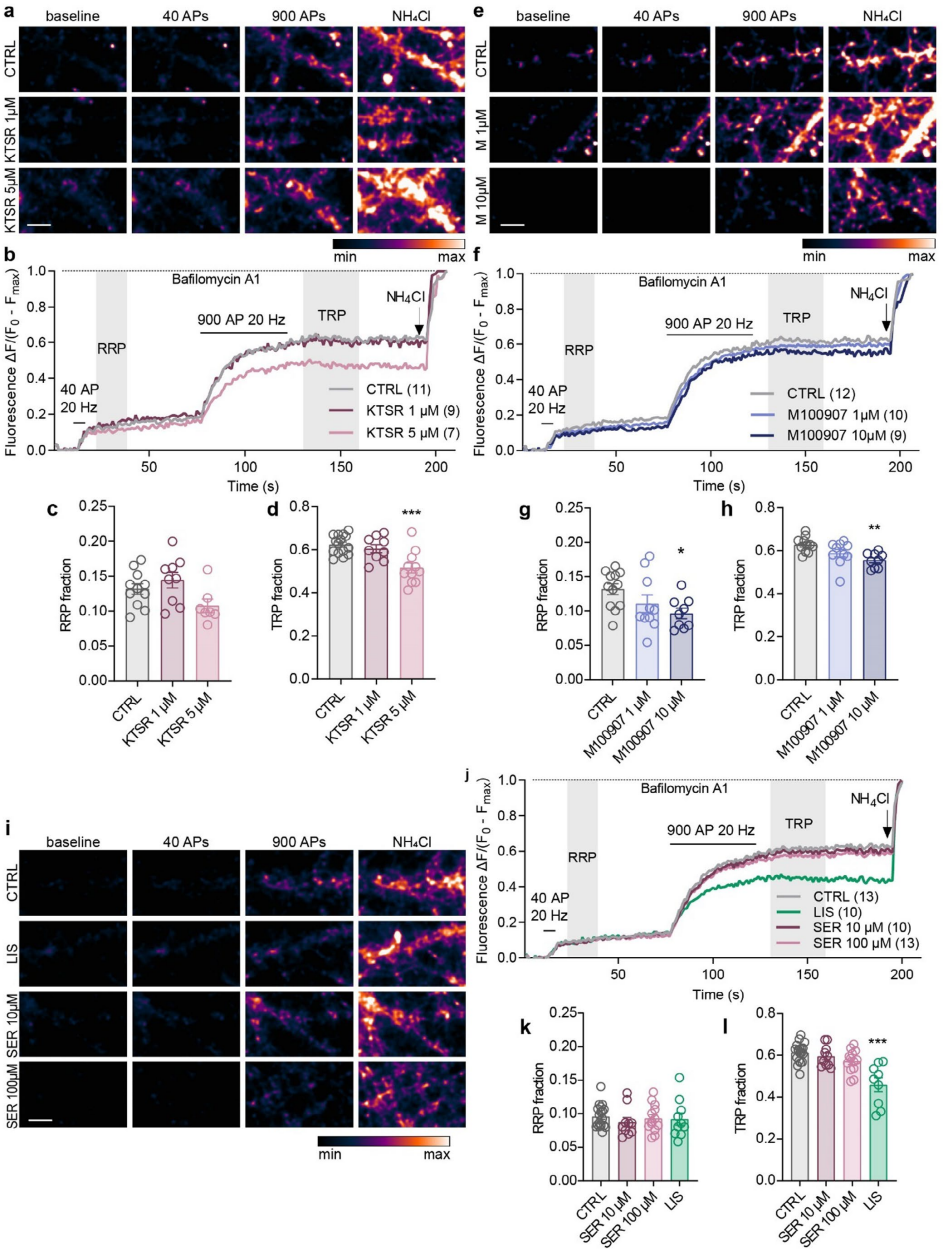
5‐HT2AR antagonists ketanserin and M100907, as well as the nonselective 5‐HT2AR agonist lisuride, affect SV pools. Representative colour gradient images (range shown in the rectangle below) from live‐cell imaging of sypHy‐expressing neurons treated with vehicle (CTRL) or ketanserin (KTSR) 1 or 5 μM (a), CTRL or M100907 1 or 10 μM (e), and CTRL, serotonin (SER) 10 or 100 μM or lisuride 10 μM (i). Images show cells during baseline recording, stimulation with 40 and 900 APs at 20 Hz and upon NH_4_Cl pulse, in the presence of bafilomycin A1. Scale bar is 10 μM. (b, f, j) Average traces of sypHy fluorescence intensity normalised to minimum (baseline, *F*_0_) and maximum (NH_4_Cl pulse, *F*_max_). Total number of recordings per treatment from 3 independent culture preparations is indicated in brackets. Quantification of the RRP (c, g, k) and TRP (d, h, l) fractions of SVs from the respective experiments. In graphs, bars show the mean, whiskers SEM and circles all data points. Statistical significance was assessed by one‐way ANOVA (KTSR: RRP: *F*_(2,24)_ = 3.294, *p* = 0.054, TRP: *F*_(2,24)_ = 21.59, *p* < 0.001; M100907: RRP: *F*_(2,28)_ = 3.476, *p* = 0.045, TRP: *F*_(2,28)_ = 4.757, *p* = 0.017; SER + LIS: RRP: *F*_(3,48)_ = 0.353, *p* = 0.787, TRP: *F*_(3,48)_ = 16.12, *p* < 0.001) with Dunnett's multiple comparisons test CTRL versus each drug (KTSR: TRP: 1 μM *p* = 0.676, 5 μM *p* < 0.001; M100907: RRP: 1 μM *p* = 0.212, 10 μM *p* = 0.029, TRP: 1 μM *p* = 0.242, 10 μM *p* = 0.010; SER + LIS: TRP: SER 10 μM *p* = 0.829, SER 100 μM *p* = 0.189, LIS *p* < 0.001).

It has been proposed that the acute effects of psychedelics on perception, cognition and behaviour are dependent on their action as partial agonists of the 5‐HT2AR (Hesselgrave et al. 2021; Holze, Avedisian, et al. 2021; Ly et al. 2018; Odland et al. 2022; Preller et al. 2017). To test an effect of modulating the activity of 5‐HT2AR on SV fusion competence, we applied potent antagonists of 5‐HT2AR, ketanserin and M100907. First, we applied 1 and 5 μM ketanserin, a 5‐HT2A antagonist previously shown to block behavioural and cellular effects of psychedelics (Holze, Vizeli, et al. 2021; Ly et al. 2018; Preller et al. 2017; Vollenweider et al. 1998). While 1 μM ketanserin had no effect, 5 μM ketanserin reduced TRP but not RRP when assessed 45 min after the application (Figure 4a–d; RRP: CTRL 0.13 ± 0.03, 1 μM 0.14 ± 0.03, 5 μM 0.11 ± 0.03; TRP: CTRL 0.62 ± 0.05, 1 μM 0.61 ± 0.06, 5 μM 0.48 ± 0.04). To further confirm the specificity of these results, we tested another 5‐HT2AR antagonist, M100907. This compound exhibits higher selectivity towards the 2A receptor than ketanserin that additionally affects adrenergic and histamine receptors (PDSP 2023; Roth et al. 2000). While 1 μM M100907 had no effect on synaptic vesicle pools, both RRP and TRP were significantly reduced in cells treated with 10 μM M100907 for 45 min (Figure 4e–h; RRP: CTRL 0.13 ± 0.03, 1 μM 0.11 ± 0.04, 10 μM 0.10 ± 0.02; TRP: CTRL 0.62 ± 0.05, 1 μM 0.60 ± 0.03, 10 μM 0.56 ± 0.03). The fact that psychedelics acting by partial agonism of 5‐HT2AR had a similar effect on the RRP and TRP as established 5‐HT2AR antagonists was unexpected and hampered the use of ketanserin or M100907 in combination with psychedelics to test the involvement of 5‐HT2AR activation in psychedelic‐induced changes in SV fusion competence.

Next, we applied serotonin, which acts as the physiological agonist of the 5‐HTRs, and lisuride, a substance that has previously been utilised in studies as a non‐hallucinogenic counterpart of LSD (Egan et al. 1998; González‐Maeso et al. 2003, 2007; Pieri et al. 1978). Lisuride functions as a (partial) agonist at the 5‐HT2A and other serotonergic receptors with the exception of 5‐HT2B, and also displays relatively high affinity to dopamine and adrenergic receptors. While serotonin applied at 10 or 100 μM concentration had no effect on synaptic vesicle pools, 10 μM lisuride significantly reduced the TRP, but not the RRP (Figure 4i–l; RRP: CTRL 0.10 ± 0.02, SER10 0.09 ± 0.02, SER100 0.09 ± 0.02, LIS 0.09 ± 0.03; TRP: CTRL 0.61 ± 0.05, SER10 0.59 ± 0.05, SER100 0.57 ± 0.05, LIS 0.45 ± 0.10).

**FIGURE 5:**
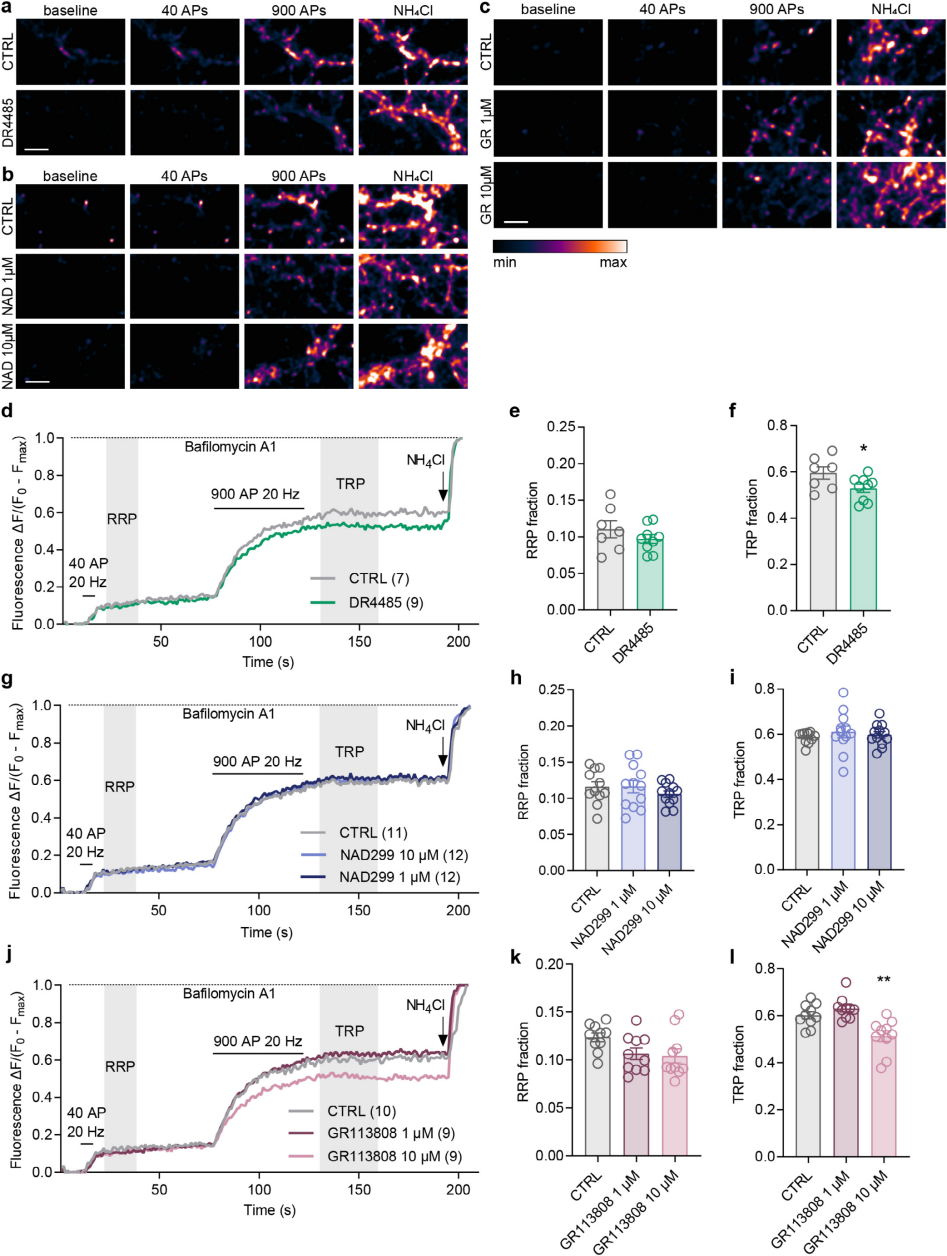
Specific 5‐HT4R and 5‐HT7R antagonists reduce the size of TRP of SVs, while the 5‐HT1AR antagonist does not affect SV fusion competence. Representative colour gradient images from live‐cell imaging of sypHy‐expressing primary cortical cultures treated with vehicle (CTRL) or 5‐HT7R antagonist DR‐4485 (1 μM; a), CTRL or 5‐HT1AR antagonist NAD299 (1 or 10 μM; b), and CTRL or 5‐HT4R antagonist GR113808 (1 or 10 μM; c) for 45 min during baseline recording, stimulation with 40 and 900 APs at 20 Hz and upon NH_4_Cl pulse, in the presence of bafilomycin A1. Scale bar 10 μM. (d, g, j) Average traces of sypHy fluorescence intensity from the respective experiments normalised to minimum (baseline, *F*_0_) and maximum (NH_4_Cl pulse, *F*_max_). Total number of recordings per treatment from 2 to 4 independent culture preparations is indicated in brackets. Quantification of the RRP (e, h, k) and TRP (f, i, l) fractions of SVs. In graphs, bars show the mean, whiskers SEM and circles all data points. Statistical significance was assessed by unpaired *t*‐test (DR4485: RRP: *t*_(14)_ = 1.098, *p* = 0.291, TRP: *t*_(14)_ = 2.160, *p* = 0.049), and one‐way ANOVA (GR113808: RRP: *F*_(2,27)_ = 3.065, *p* = 0.063, TRP: *F*_(2,27)_ = 11.41, *p* < 0.001; NAD299: RRP: *F*_(2,32)_ = 0.711, *p* = 0.499, TRP: *F*_(2,32)_ = 0.405, *p* = 0.670) with Dunnett's multiple comparisons test CTRL versus each drug (GR113808: TRP: 1 μM *p* = 0.421, 10 μM *p* = 0.004).

Given that LSD, PSI and DMT also modulate other classes of 5‐HT receptors (Halberstadt et al. 2011; Ray 2010; Tylš et al. 2023), we asked whether modulation of further receptors might also affect SV fusion competence. To find the most abundant 5‐HT receptors expressed in our experimental system, we mapped the expression of subtypes of the 5‐HT receptor using qRT‐PCR with specific primers on total RNA isolated from cultures at DIV21. Subsequent analysis revealed a moderate expression level (cycle threshold (Ct) < 30) for *Htr1a/b/d/f/, Htr2c, Htr4, Htr5a* and *Htr7* genes, and a lower level of expression (Ct > 30) for *Htr2a/b, Htr3a/b, Htr5b, and Htr6* genes as compared to the Ct values of reference genes *Gapdh* and *Actb* (Figure S1). Ct values obtained were < 35 for all receptor subtypes tested, indicating their expression in the primary cortical cultures containing both neurons and glial cells. We proceeded with testing the effects of modulation of receptor subtypes 5‐HT1A, 5‐HT4 and 5‐HT7. We observed that 1 μM DR4485, a 5‐HT7R antagonist, significantly decreased the TRP of SVs while having no effect on the RRP (Figure 5a,d–f; RRP: CTRL 0.11 ± 0.03, 0.10 ± 0.02; TRP: CTRL 0.60 ± 0.07, DR4485 0.53 ± 0.05). GR113808, an antagonist of the 5‐HT4 receptor, had a similar effect on the TRP without affecting the RRP at the 10 μM concentration, and showed no effect at 1 μM (Figure 5c,j–l; RRP: CTRL 0.12 ± 0.01, 1 μM 0.11 ± 0.02, 10 μM 0.10 ± 0.02; TRP: CTRL 0.60 ± 0.05, 1 μM 0.63 ± 0.05, 10 μM 0.51 ± 0.07). The 5‐HT1AR antagonist NAD299 had no effect on SV pools at 1 or 10 μM concentration (Figure 5b,g–I; RRP: CTRL 0.12 ± 0.02, 1 μM 0.12 ± 0.03, 10 μM 0.11 ± 0.02; TRP: CTRL 0.59 ± 0.03, 1 μM 0.61 ± 0.09, 10 μM 0.60 ± 0.05).

This pharmacological approach revealed that the 5‐HT agonist lisuride reduced the TRP similarly to the 5‐HT2A antagonists ketanserin, the 5‐HT7R antagonist DR4485, and the 5‐HT4R antagonist GR113808 (10 μM). Along the same line, the 5‐HT2AR antagonist M100907 (10 μM) decreased both the RRP and TRP, which is surprisingly similar to the effects elicited by LSD and psilocin. The only substances tested that act on receptors relevant to psychedelics' action and did not affect the SV pools were serotonin and the 5‐HT1AR antagonist NAD299. Thus, these experiments revealed complex effects of modulation of 5‐HT receptor activity on SV pools, making the interpretation of pharmacological approaches highly challenging.

### 3.4 Psilocin Reduces Paired‐Pulse Ratio Indicating Increased Probability of Glutamate Release

**FIGURE 6:**
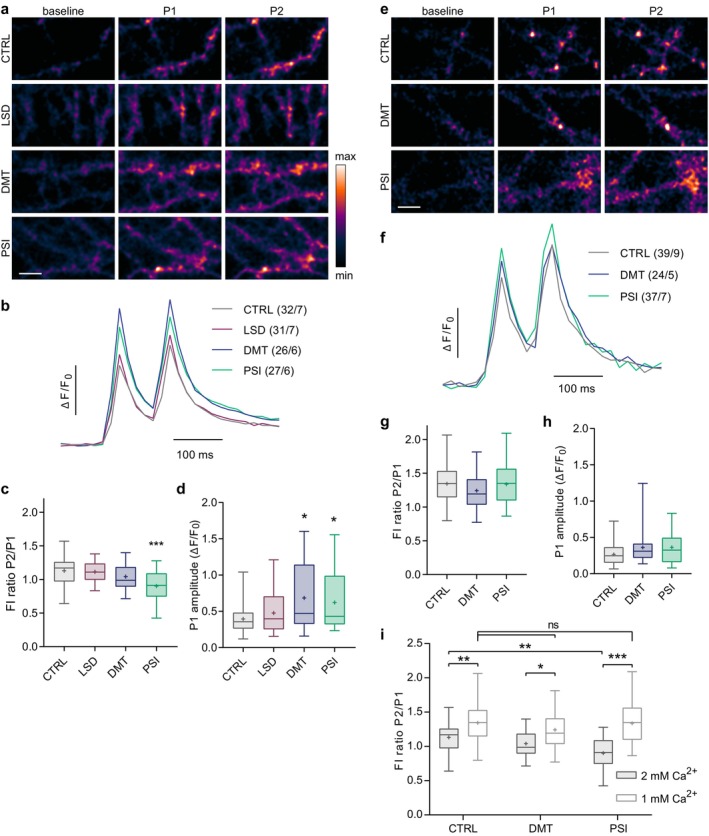
Psilocin induces paired‐pulse depression by increasing glutamate release probability. (a, e) Live‐cell imaging of mature cortical cultures expressing glutamate sensor iGluSnFR was conducted 30 min after treatment with substances. Representative colour gradient (range is shown in the rectangle) images were acquired during baseline and paired stimuli (peak 1 and 2; P1, P2) at 10 Hz in Tyrode's buffer with standard, 2 mM Ca^2+^ (a), or low, 1 mM Ca^2+^ (e). Scale bars are 10 μm. (b, f) Average traces of iGluSnFR fluorescence intensity shown as change in fluorescence (ΔF) divided by baseline fluorescence (*F*_0_). In brackets indicated per treatment: total number of recordings/numbers of independent coverslips from which these were taken; all from 3 independent culture preparations. (c, g) Fluorescence intensity (FI) ratio of P2/P1 (paired‐pulse ratio). Significance was assessed by one‐way ANOVA (c: *F*_(3,107)_ = 7.197, *p* < 0.001; g: *F*_(2,95)_ = 1.144, *p* = 0.323) with Dunnett's multiple comparisons test of CTRL versus each drug (c: LSD *p* = 0.982, DMT *p* = 0.252, PSI *p* < 0.001) in either 2 mM (c) or 1 mM (g) Ca^2+^. Amplitude of fluorescence at P1 shown as Δ*F*/*F*_0_ in 2 mM Ca^2+^ (d) or 1 mM Ca^2+^ (h). Data did not pass Shapiro–Wilk test for normality; thus, significance was assessed by Kruskal –Wallis test (d: *H*_(3,116)_ = 9.183 *p* = 0.027, h: *H*_(2,99)_ = 4.395, *p* = 0.111) with Dunn's multiple comparisons test of CTRL versus each drug (d: LSD *p* > 0.999, DMT *p* = 0.037, PSI *p* = 0.048). (i) Comparison of paired‐pulse ratios in standard (2 mM; c) and low (1 mM; g) Ca^2+^ concentration. Significances were assessed by two‐way ANOVA (drug effect: *F*_(2,177)_ = 4.055, *p* = 0.019; Ca^2+^ effect: *F*_(1,177)_ = 56.86, *p* < 0.001; interaction: *F*_(2,177)_ = 4.237, *p* = 0.016) with Šídák's multiple comparisons test for the differences between 1 mM and 2 mM Ca^2+^ per treatment (CTRL *p* = 0.007, DMT *p* = 0.015, PSI *p* < 0.001).

The following experiments aimed to map the effects of psychedelics on presynaptic properties in more detail. To assess the effect of psychedelics on evoked glutamate release, we utilised a genetically encoded glutamate sensor SF‐iGluSnFR.S72A (referred to as iGluSnFR). This sensor rapidly increases its fluorescence upon binding to glutamate and allows the detection of brief synaptic glutamate transients evoked by a single field stimulus (Marvin et al. 2013, 2019). Mature cortical cultures expressing iGluSnFR were treated with drugs (LSD, DMT, PSI) or vehicle (CTRL) for 30 min, and glutamate release evoked by stimulation with a pair of stimuli applied at 10 Hz was monitored (Figure 6a,b). Paired stimulation enables the assessment of evoked glutamate transients (amplitude of first peak, P1) as well as paired‐pulse ratio (PPR, ratio P2/P1), which is commonly used as a readout for short‐term presynaptic plasticity (Regehr 2012). The PPR was decreased in cells treated with PSI as compared to CTRL, while LSD and DMT had no effect (Figure 6a–c; CTRL1.13 ± 0.23, LSD 1.11 ± 0.16, DMT1.04 ± 0.19, PSI0.90 ± 0.23). A decline in the PPR is typically ascribed to an increase in synaptic release probability. In accordance with this supposition, we observed increased glutamate release by 50% as compared to controls in cells treated with PSI. Surprisingly, the evoked glutamate release was increased by 70% also in DMT‐treated cells (Figure 6b,d). To corroborate this observation, we repeated the same experiment with the extracellular calcium concentration reduced to 1 mM (Figure 6e,f). A reduction in extracellular calcium levels has been demonstrated to reduce the release probability, increasing the paired‐pulse facilitation of release (Oleskevich et al. 2000; Zucker and Regehr 2002). In accordance with this assumption, the paired‐pulse ratio and the amplitudes of the first glutamate transient were unchanged in PSI‐treated cells in a buffer with low [Ca^2+^] (Figure 6g,h). Finally, when we directly compared the paired‐pulse ratio in physiological (2 mM) and low (1 mM) [Ca^2+^], we confirmed a significant increase in the PPR in the low [Ca^2+^] compared to the standard [Ca^2+^] in all groups (Figure 6i; PPR in 2 mM and 1 mM Ca^2+^: CTRL 1.13 ± 0.23 and 1.34 ± 0.27, DMT 1.04 ± 0.19 and 1.24 ± 0.26, PSI 0.90 ± 0.23 and 1.34 ± 0.29). This is in line with the key role of calcium build‐up in the paired‐pulse facilitation (Regehr 2012).

In conclusion, DMT and PSI treatment resulted in an enhancement of evoked glutamate release upon individual stimuli, indicating their effect on glutamate release. Moreover, PSI, but not DMT, induced paired‐pulse depression in mature cortical neurons, indicating its effect on the short‐term plasticity of glutamatergic neurotransmission.

### 3.5 LSD And PSI Decrease Presynaptic Calcium Concentration During Prolonged Depolarization

**FIGURE 7:**
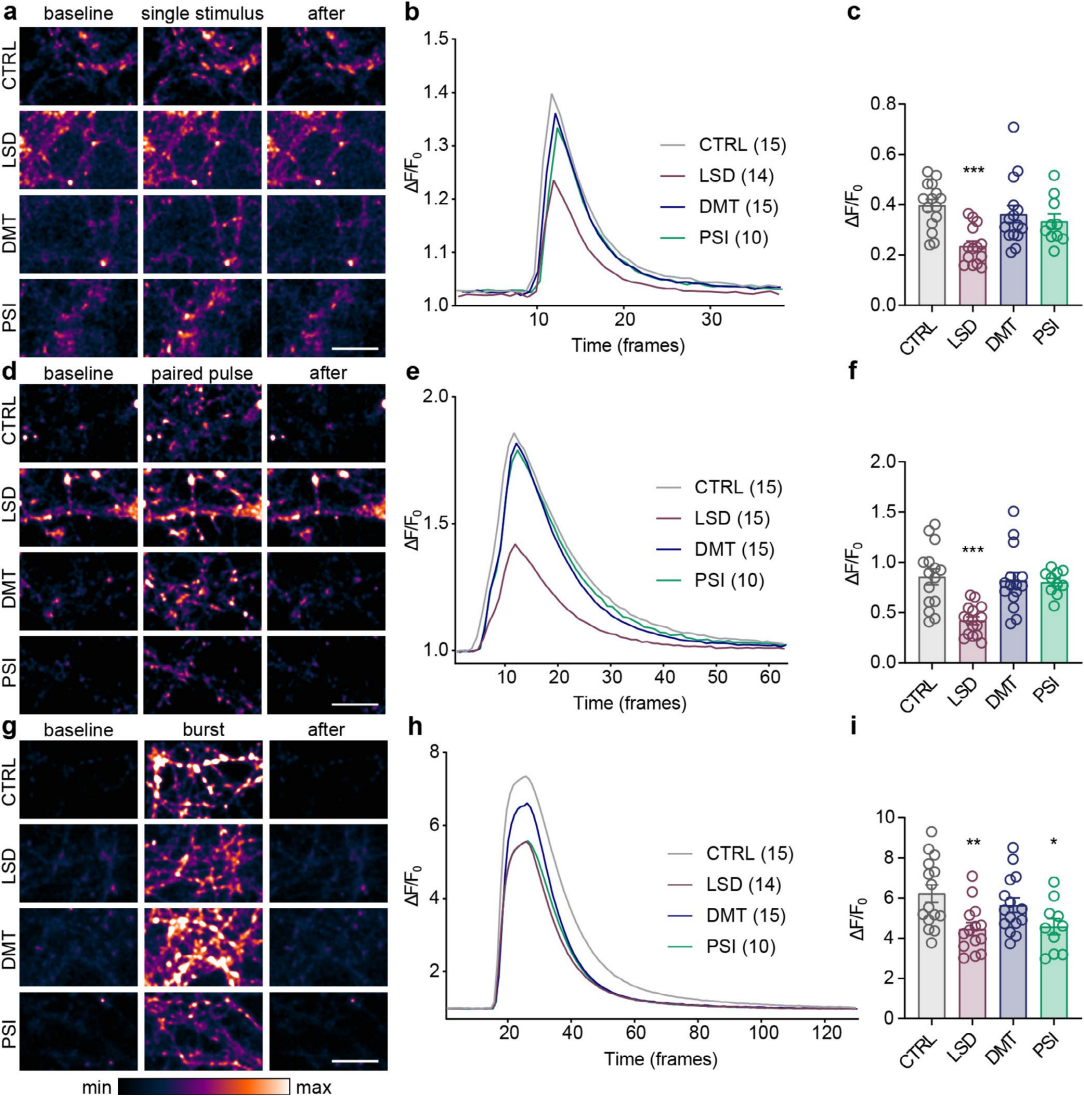
LSD and PSI reduce evoked presynaptic calcium transients. Representative colour gradient (range is shown in the rectangles below) images from live‐cell imaging of mature cortical cultures expressing calcium indicator synGCaMP6, treated with psychedelics for 30 min, during baseline, stimulus (a single stimulus, d paired‐pulse, g burst) and after the stimulation. Scale bars 10 μm. (b, f, h) Average traces of synGCaMP6 fluorescence intensity from respective experiments shown as change in fluorescence (Δ*F*) divided by baseline fluorescence (*F*_0_). Total number of recordings from 3 independent experiments indicated in brackets. Quantification of the amplitude of fluorescence intensity (as Δ*F*/*F*_0_) following the delivery of single stimulus (c), paired‐pulse (f) and burst of stimuli (30 APs; i). In graphs, bars show the mean, whiskers SEM and circles all data points. Statistical significance was assessed by one‐way ANOVA (single stimulus: *F*_(3,50)_ = 6.775, *p* < 0.001; paired‐pulse: *F*_(3,51)_ = 10.26, *p* < 0.001; burst: *F*_(3,50)_ = 4.824, *p* = 0.005) with Dunnett's multiple comparisons test for CTRL versus each drug (single stimulus: LSD *p* < 0.001, DMT *p* = 0.671, PSI *p* = 0.296; paired‐pulse: LSD *p* < 0.001, DMT *p* = 0.946, PSI *p* = 0.910; burst: LSD *p* = 0.005, DMT *p* = 0.577, PSI *p* = 0.019).

Given the observed alterations in the SV fusion competence and paired‐pulse ratio, we decided to assess the effect of psychedelics on evoked presynaptic calcium influx, which is the first step in the evoked glutamate release. We utilised the genetically encoded calcium indicator synGCaMP6, which is fused to synaptophysin, resulting in its exclusive localization to the presynaptic endings (Müller et al. 2022). As previously, we treated mature cortical cultures with psychedelics or vehicle for 30 min before live‐cell imaging. We monitored presynaptic calcium transients evoked by single or paired stimuli applied at 10 Hz and a burst of 30 stimuli at 40 Hz. LSD treatment significantly decreased synGCaMP6 fluorescence across all three protocols indicating a robust LSD‐induced reduction of evoked presynaptic calcium transients (Figure 7a–i; single stimulus: CTRL 0.40 ± 0.09, LSD 0.23 ± 0.07; paired stimuli: CTRL 0.86 ± 0.31, LSD 0.42 ± 0.15; burst: CTRL 6.21 ± 1.71, LSD 4.47 ± 1.19). PSI treatment decreased only the burst‐induced presynaptic calcium transients (Figure 7g–i, single stimulus: PSI 0.33 ± 0.09; paired stimuli: PSI 0.80 ± 0.12; burst: PSI 4.58 ± 1.27). DMT treatment did not induce any observable changes in presynaptic calcium transients (single stimulus: DMT 0.36 ± 0.13; paired stimuli: DMT 0.82 ± 0.30; burst: DMT 5.65 ± 1.41). Thus, these experiments revealed a specific effect of psychedelics on evoked presynaptic calcium transients in cultured cortical neurons. Moreover, since calcium transients evoked by single or paired stimuli did not differ between PSI and controls, we conclude that the PSI‐induced short‐term plasticity cannot be attributed to the changes in evoked calcium influx.

## 4 Discussion

This study shows that the serotonergic psychedelics LSD, DMT and psilocin specifically modulate distinct aspects of glutamatergic neurotransmission, including fusion competence of synaptic vesicles, evoked presynaptic calcium transients, glutamate release, and short‐term plasticity in rat cortical cultures. The substance‐specific effects on neurotransmission are summarised in Table 1. We used a 10 μM concentration for LSD and psilocin, and 90 μM for DMT in all experiments. These concentrations have been previously validated by Ly et al. (2018) as the most effective in inducing structural neuroplasticity in primary neuronal cultures. These concentrations also induced a robust increase in nuclear pCREB 24 h after treatment, which is consistent with the previously published data showing a drug‐induced expressional reprogramming that is believed to drive the long‐term effects of psychedelics in vivo (Calder and Hasler 2023; Fuente Revenga et al. 2021).

**TABLE 1:** Summary of effects of serotonergic psychedelics LSD, psilocin and DMT on presynaptic parameters tested in this study.

	LSD	PSI	DMT
RRP	↓	↓	—
TRP	↓	↓	↓
Ca^2+^ concentration (1 AP)	↓	—	—
Ca^2+^ concentration (30 AP)	↓	↓	—
Glutamate amplitude (1 AP)	—	↑	↑
Paired‐pulse ratio	—	↓	—

All psychedelics rapidly reduced the TRP of SVs, while LSD and psilocin also reduced the RRP of SVs. From a functional perspective, the reduction in the RRP directly affects the release of neurotransmitters in response to a brief series of stimuli. Conversely, reduction in the TRP impacts neurotransmitter release upon prolonged stimulation, because TRP serves as the source pool for replenishing SVs to the RRP under conditions of moderate sustained physiological activity (Alabi and Tsien 2012). Using imaging of presynaptic calcium sensors, we observed changes in evoked presynaptic transients in cells treated with LSD and psilocin. While LSD reduced the presynaptic calcium transients evoked by single and paired stimuli, as well as calcium levels during burst stimulation, psilocin affected only responses to burst stimulation. Changes in the presynaptic calcium transients evoked by single stimuli are mainly attributable to changes in calcium influx via vltage‐gated calcium channels and modification of cytoplasmic calcium buffers. Additionally, changes in calcium clearance or calcium‐dependent calcium release form internal stores affect the kinetics of calcium transients especially during burst stimulation. Changes in calcium influx were linked to the regulation of RRP (Thanawala and Regehr 2013), which is in agreement with the reduction in RRP measured in LSD and psilocin‐treated cells in this study. Thus, this data revealed changes in presynaptic calcium influx and/or homeostasis, as another presynaptic mechanism modulated by LSD and psilocin. Interestingly, both DMT and psilocin increased evoked glutamate transients, indicating their effect on presynaptic release probability. Psilocin additionally reduced PPR, the readout for the short‐term plasticity of neurotransmission. The psilocin‐specific decrease in PPR could arise from an increase in the probability of evoked glutamate release and concomitant decrease in the RRP size, both rendering synaptic responses to repetitive stimulation more prone to a short‐term depression. Collectively, our data indicate that psychedelics dampen glutamate release by modulating multiple presynaptic properties. This is in line with work by Hu et al. that reported depressed firing of cultured cortical neurons following exposure to DOI, another psychedelic drug (Hu et al. 2016). On the systemic level, psychedelics decrease the power of electrophysiological signals resulting from desynchronization of brain activity and decreased functional network connectivity (Gattuso et al. 2022; Muthukumaraswamy et al. 2013). How these systemic effects align with reduction of glutamatergic neurotransmission remains to be addressed by further investigations.

Interestingly, the temporal profile of the effects on synaptic vesicle pools differed for the psychedelics tested in this study. LSD elicited a significant and consistent reduction in the RRP and TRP following both acute and 30 min treatment. The effect of PSI on SV fusion competence became discernible only after 30 min of treatment. Conversely, DMT decreased the TRP only after acute treatment. The observed variability in the temporal profile and affected SV pools could arise from the specific binding affinities of these drugs for serotonergic receptors, and in particular their selectivity towards the 5‐HT2A. While the binding affinity of LSD towards 5‐HT2AR is in the low nanomolar range, that of psilocin is in the order of tens and DMT in the hundreds of nM (PDSP 2023). We have at least partially considered this fact and used different concentrations of the drugs (see above). The higher affinity of LSD compared to psilocin might contribute to the slightly different temporal profiles of observed effects for these drugs applied in equimolar concentration in this study. The short‐lasting effect of DMT (evident after 2–5 min but not 30 min later) might result from its rapid metabolism. In vivo, DMT is rapidly metabolised and induces a transient psychedelic state lasting up to 30 min or 1 h in human (Riba et al. 2015; Strassman et al. 1994; Szára 1956). The transcript and specific activity of the enzyme necessary for the metabolism of DMT, monoamine oxidase, was detected in the primary cultures analogous to those used in this study (Chaudhuri et al. 2013; Maher and Davis 1996; Ostadkarampour and Putnins 2021). Thus, it is feasible that the observed transient effect of DMT on the TRP is due to the limited half‐life of this compound in our experimental system. Of note, the effect of drugs on SV fusion competence that were evident within minutes disappeared 24 h later. It will be interesting to test in future studies whether the initial synaptic effects of psychedelics contribute to the activation of signalling cascades leading to the persistent changes in neuronal gene expression.

In contrast to the effects observed with serotonergic psychedelics mainly acting as partial agonists of 5‐HT2A, application of the physiological ligand of these receptors, serotonin, did not affect SV fusion competence. This is in agreement with distinct behavioural, cellular and molecular effects described in a number of previous studies for psychedelics and non‐psychedelic 5‐HTR agonists (González‐Maeso et al. 2007; Ly et al. 2018; Vargas et al. 2023). This can be explained by the phenomenon of biased agonism, that postulates differential activation of downstream signalling cascades by distinct ligands of the same 5‐HTR (Berg et al. 1998; Kossatz et al. 2024; Martí‐Solano et al. 2015). Vargas and co‐authors recently suggested that the specific receptor localization can further contribute to this phenomenon (2023). They demonstrated that psychedelics, as lipophilic substances, induce their neuroplastic effects by activating a specific population of intracellular 5‐HT2A receptors, which are not accessible to serotonin or other membrane‐impermeable 5‐HT2A agonists (Vargas et al. 2023).

As a result of biased agonism, psychedelics induce the Gα_i/o_ signalling pathway, apart from the canonical 5‐HT2AR‐coupled Gα_q/11_ pathway, which distinguishes them from the physiological 5‐HT2A ligand serotonin and other non‐psychedelic 5‐HT2AR agonists (González‐Maeso et al. 2007; Kossatz et al. 2024). The activation of Gα_i/o_ by psychedelics LSD and DOI was also shown to depend on the interaction of 5‐HT2AR with metabotropic glutamate receptor mGluR2 (González‐Maeso et al. 2008), and mGluR2 was demonstrated to be necessary for the pharmacological effects of DOI to take place (Moreno et al. 2011). mGluR2 is found mostly at the presynaptic boutons and preterminal axons (Bodzęta et al. 2021; Jin et al. 2018), where it regulates transmission by inhibition of glutamate release from the presynapse via Gα_i/o_ signalling (Nicholls et al. 2006). Specifically, the Gα_i/o_ pathway inhibits the adenylyl cyclase activity, leading to reduced cAMP production and therefore lower activity of cAMP‐dependent protein kinase (PKA), the main regulator of presynaptic release probability. One of the target proteins of PKA is synapsin 1, the main regulator of SV clustering and fusion competence (Menegon et al. 2006). Another target of PKA is SNAP‐25, a constituent of the SNARE complex, which mediates the fusion of SVs with the plasma membrane. Its PKA‐dependent SNAP‐25 phosphorylation reduces the stability of the SNARE complex and increases the size of RRP (Nagy et al. 2004). Thus, the dampening of cAMP signalling downstream of psychedelics‐induced activation of 5‐HT2A Gα_i/o_ could explain changes in SV pools and release probability observed in our experiments. Finally, the signalling via Gβγ subunits leads to hyperpolarisation‐induced decrease in firing rates via activation of G‐protein‐coupled inward rectifying potassium channels (GIRK) and reduction in calcium influx via direct inhibition of the voltage‐gated calcium channels (Altier 2012; Dolphin 2003; Qin et al. 1997). Thus, psychedelics‐induced signalling via Gα_i/o_ and Gβγ downstream of the 5‐HT2A/mGluR2 complex is compatible with the decrease in the probability of SV fusion and in calcium transients as we observed with LSD, psilocin, and partially also with DMT. However, substance‐specific mechanisms, possibly involving further interaction with other receptors (including non‐serotonergic) or signalling pathways, need to be addressed by future studies.

The complex ligand‐specific signaling by 5‐HTRs could also explain the unexpected effects of well‐established 5‐HT2AR, 5‐HT4R and 5‐HT7R antagonist, which were similar to the effects of psychedelics. The fact that the antagonists show an effect in (presumable) absence of any agonist indicate a certain baseline activity of the receptors in our experimental system. Indeed, constitutive activity has been proposed for multiple 5‐HTR subtypes, sometimes with contrasting results from in vitro and in vivo models. Specifically, constitutive activity was demonstrated in the case of the 5‐HT2AR, 5‐HT4R and 5‐HT7R (Berg et al. 2005; Harvey 2003; Kvachnina et al. 2009; Mialet et al. 2000; Ponimaskin et al. 2002). The antagonists used in this study, including ketanserin and M100907 (5‐HT2A) and GR113808 (5‐HT4), were previously shown to act as inverse agonists in vitro (Egan et al. 1998; Mialet et al. 2000; Weiner et al. 2001). Thus, the effects of antagonists observed in this study could arise from a reduction of 5‐HTRs baseline activity and its corresponding signaling: Gα_s_ in the case of 5‐HT4 and 5‐HT7 and Gα_q/11_ in the case of 5‐HT2A.

## Author Contributions

**Aneta Petrušková:** conceptualization, investigation, funding acquisition, writing – original draft, methodology, validation, visualization, data curation, formal analysis, project administration. **Debarpan Guhathakurta:** supervision, methodology. **Enes Yağız Akdaş:** methodology, resources. **Bartomeu Perelló‐Amorós:** resources, methodology. **Renato Frischknecht:** funding acquisition, methodology, resources. **Eva‐Maria Weiss:** funding acquisition, project administration. **Tomáš Páleníček:** supervission, funding acquisition, resources. **Anna Fejtová:** writing – review and editing, supervision, resources, conceptualization, methodology, funding acquisition.

## Conflicts of Interest

Tomáš Páleníček declares to have shares in ‘Psyon s.r.o.’, has founded ‘PSYRES–Psychedelic Research Foundation’, and has shares in ‘Společnost pro podporu neurovědního výzkumu s.r.o.’ T.P furthermore reports consulting fees from GH Research and CB21‐Pharma outside the submitted work. T.P is involved in clinical trials of Compass Pathways with psilocybin, MAPS trial with MDMA and GH Research trial with 5‐MeO‐DMT outside the submitted work. The other authors have no relevant financial or non‐financial interests to disclose.

### Peer Review

The peer review history for this article is available at https://www.webofscience.com/api/gateway/wos/peer‐review/10.1111/jnc.70020.

## Supporting information


Data S1.

## Data Availability

The datasets generated during the current study are available from the corresponding author on reasonable request, as well as materials used in the study.
