# Supplementary material for: Serotonergic Psychedelics Rapidly Modulate Evoked Glutamate Release in Cultured Cortical Neurons
Source: J Neurochem. 2025 Feb 28;169(3):e70020. doi: 10.1111/jnc.70020 (PMC11871419; doi:10.1111/jnc.70020)
Supplement: Supplementary file 1 — Data S1. [file JNC-169-0-s001.pdf]

Supplementary material

Serotonergic psychedelics rapidly modulate evoked glutamate release in cultured cortical neurons

Aneta Petrušková[1,2,3], Debarpan Guhathakurta[1], Enes Yağız Akdaş[1], Bartomeu Perelló-Amorós[4], Renato Frischknecht[4], Eva-Maria Weiss[1], Tomáš Páleníček[2,3], Anna Fejtová[1#]

[1] Department of Psychiatry and Psychotherapy, Universitätsklinikum Erlangen, Friedrich-Alexander-Universität Erlangen-Nürnberg, Erlangen, Germany
[2] National Institute of Mental Health, Klecany, Czech Republic
[3] Third Faculty of Medicine, Charles University, Prague, Czech Republic
[4] Department of Biology, Animal Physiology, Friedrich-Alexander-Universität Erlangen-Nürnberg, Erlangen, Germany.

a

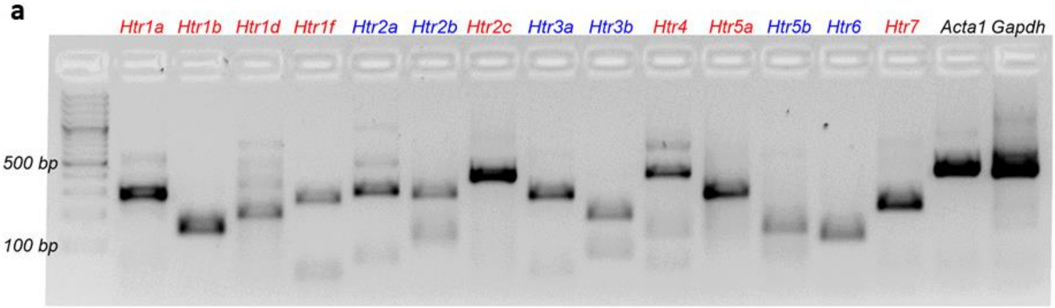

b

Gene	Ct value	Gene	Ct value
Htr1a	26.75±0.25	*Htr3b*	33.38±0.32
Htr1b	28.62±0.25	*Htr4*	27.71±0.42
Htr1d	27.69±0.19	*Htr5a*	25.89±0.22
Htr1f	26.22±0.31	*Htr5b*	32.74±0.38
Htr2a	33.49±0.21	*Htr6*	33.49±0.43
Htr2b	32.34±0.28	*Htr7*	25.34±0.12
Htr2c	24.82±0.21	*Actb*	17.49±0.05
Htr3a	30.37±0.11	*Gapdh*	16.51±0.28

Fig. S1 Expressional mapping of 5-HT receptor subtypes in rat cortical cultures. a qRT-PCR were resolved on agarose gel electrophoresis to validate expression of 5-HT receptor subtypes. The presence of distinct bands of predicted size (as compared to the DNA ladder) confirms the specificity of amplification. **b** Table shows the respective Ct values for all tested genes. Ct values<35 demonstrate the expression level of gene targets, which is positive for all 5-HT receptor subtypes. Red captions indicate genes with moderate expression (Ct < 30), while blue captions indicate lower expression of target genes (Ct > 30). Black captions of genes indicate the reference genes used in the qRT-PCR analysis. Values are given as mean ± SEM.

Supplementary methods for qRT-PCR

250 000 rat cortical neurons were seeded onto poly-L-lysin coated 12-well plate (isolation and seeding according to main methods). After 21 days of cultivation, RNA extraction was carried out using the Nucleospin RNA isolation kit (MN #740955.250, Germany) including a DNAse treatment step following the manufacturer's guidelines. 100 ng total RNA was reverse transcribed to synthesize complementary DNA (cDNA) using LunaScript® RT SuperMix Kit (NEB, #E3010, USA) according to manufacturer`s protocol. For the subsequent quantitative reverse transcription PCR (qRT-PCR) analysis, approximately 0.5 ng of RNA-containing cDNA, 0.4 µM final concentration of each primer, and Luna® Universal qPCR Master Mix (NEB, #M3003, USA) were mixed. The qPCR cycling conditions involved an initial denaturation step at 95°C for 1 minute followed by 45 cycles consisting of a 10-second denaturation at 95°C, a 20-second annealing step at 60°C, and a 30-second elongation at 68°C. All primers except *Htr1a* and *Htr1f* contain an exon/exon junction site. After amplification step, melting curves were plotted. Ct values were directly obtained using Light Cycler 480 II software. Following the qRT-PCR, the PCR amplicons were subjected to validation through electrophoresis on a 2% agarose gel.

Table S1 Primer sequences used in qRT-PCR together with their predicted amplicon size.

Gene name	Primer sequences	Amplicon length
Htr1a	CTAGCATCTCCGACGTGACC	288
	AGGTGCAGCACAGTACATCC	
Htr1b	AAGTCTGTGGCAGCGACTAA	157
	CTTCTCTCAGGTTCCCTTGTCC	
Htr1d	AGCTCAGCGGGGTCGTG	197
	GACAAAACATTCCAGTTACCAAGACTC	
Htr1f	CCTGGCCTTGATGACAACCA	261
	AACGCTATAGCCGACAGGTG	
Htr2a	TTTGAGAGGGGCTCTCTGGT	294
	AAGGCCACCGGTACCATTC	
Htr2b	GGTGGCTGATTTGCTGGTTG	277
	TGGGATGGCGATGCCTATTG	
Htr2c	TGGTCTTCGTCCGCTTAGAA	378
	GGTAATAGTTGACAACCCGCT	
Htr3a	GCACTCCCCTCATTGGTGTC	264
	GGTTTCCCATGGCTGGAAGA	
Htr3b	TGTGGTACCGAGAGGTTTGG	183
	TCGTTCCGGAAGAGTTCACG	
Htr4	CTCTGGCACAGACACCTCAG	376
	CACAGGACCCTGGGCAC	
Htr5a	CATACCTGAAGCTGTGGAGGTGA	271
	GGAGTTGGAATAGCCCAGCC	
Htr5b	CCACCTGCGGAGCTTTCTAC	140
	TGCTTCCTTTGCCTGCGTG	
Htr6	CCAACATAGCTCAGGCCGT	121
	AAGTCCCGCATAAAGAGCGG	
Htr7	ATGTCTGTGGCTGGGCTATG	217
	CACAGTGGTCAGAGTTTTGTCTTA	
Actb	CTTCGAGCAAGAGATGGCCA	413
	GCTTGCTGATCCACATCTGC	
Gapdh	GACCCCTTCATTGACCTCAACT	396
	GTCATGGATGACCTTGGCCA	
